# Succession of Microbial Communities in Waste Soils of an Iron Mine in Eastern China

**DOI:** 10.3390/microorganisms9122463

**Published:** 2021-11-29

**Authors:** Qin Zhang, Pengfei Wei, Joseph Frazer Banda, Linqiang Ma, Weiao Mao, Hongyi Li, Chunbo Hao, Hailiang Dong

**Affiliations:** 1 School of Water Resources and Environment, China University of Geosciences, Beijing 100083, China; zhangqin0908@126.com (Q.Z.); esepengfei@163.com (P.W.); jofrazer@gmail.com (J.F.B.); linqiangma@outlook.com (L.M.); mmmaoweiao@126.com (W.M.); hongyili2021@163.com (H.L.); 2 Geomicrobiology Laboratory, State Key Laboratory of Biogeology and Environmental Geology, China University of Geosciences, Beijing 100083, China; Dongh@miamioh.edu; 3 Department of Geology and Environmental Earth Science, Miami University, Oxford, OH 45056, USA

**Keywords:** mine soils, ecological restoration, microbial community succession, acid mine drainage, pH, *Acidobacteriota*

## Abstract

The reclamation of mine dump is largely centered on the role played by microorganisms. However, the succession of microbial community structure and function in ecological restoration of the mine soils is still poorly understood. In this study, soil samples with different stacking time were collected from the dump of an iron mine in China and the physicochemical characteristics and microbial communities of these samples were comparatively investigated. The results showed that the fresh bare samples had the lowest pH, highest ion concentration, and were the most deficient in nutrients while the acidity and ion concentration of old bare samples decreased significantly, and the nutritional conditions improved remarkably. Vegetated samples had the weakest acidity, lowest ion concentration, and the highest nutrient concentration. In the fresh mine soils, the iron/sulfur-oxidizers such as *Acidiferrobacter* and *Sulfobacillus* were dominant, resulting in the strongest acidity. Bacteria from genera *Acidibacter*, *Metallibacterium*, and phyla *Cyanobacteria*, WPS-2 were abundant in the old bare samples, which contributed to the pH increase and TOC accumulation respectively. *Acidobacteriota* predominated in the vegetated samples and promoted nutrient enrichment and plant growth significantly. The microbial diversity and evenness of the three types of soils increased gradually, with more complex microbial networks, suggesting that the microbial community became more mature with time and microorganisms co-evolved with the mine soils.

## 1. Introduction

Large-scale opencast metal mining activities can cause great degradation to ecosystems [1], although this modern mining technology is an efficient and cost-effective mode for the exploitation of mineral resources. In the process of opencast mining, massive stripped soils and rocks are discharged, which cover much land and destroy natural vegetation. Mine soils are usually characterized by poor soil structure, high bulk density, low nutrient availability, low structural stability, and low biomass productivity [2,3]. Furthermore, sulfur-containing minerals (e.g., pyrite (FeS_2_)), which are very common in mining wastes, can be oxidized and generate acid mine drainage (AMD) in a humid area, which usually has extremely low pH and elevated concentrations of metals as well as metalloids, posing a severe threat to our ecosystem [4]. Thereby, ecological restoration of mine dumps has become an urgent and arduous task to minimize the risk of land degradation and resident health.

The reclamation of the mine dump is largely centered on the role played by microorganisms. Their ecological functions in the soils are immense as they act as biofertilizers and degraders of organic matter [5]. However, compared with the physicochemical characteristics, the microbial properties of mine soils are relatively underexplored and are still not well understood [6,7]. Furthermore, the ultimate goal of ecological restoration of dump sites is to reestablish a productive, sustainable, and functional vegetation. The vegetation restoration of mine soils is a long-term process and in the various stages, and different microbial groups play important roles in driving nutrient cycling, improving soil quality, promoting plant growth, and sustaining ecological balance in mining areas. Therefore, understanding the succession process of microbial community structure and function from fresh mine soils to vegetated mine soils can offer us scientific guidance to manage land reclamation and ecological reconstructions of mining areas.

However, because the natural recovery process usually takes several decades, or even hundreds of years [8], it may be impossible for researchers to conduct continuous monitoring on this time scale. Therefore, a chrono-sequence approach, in which sites of different ages were assumed to represent points in time in the succession of individual sites, was developed and has effectively been used to examine the effect of time on soil development, ecological succession, and vegetation recovery following disturbance [9,10,11]. However, this method is mostly used to study the soil ecological restoration process in coal mining areas [12], and there is very little research on the metallic mine dumps widely distributed across the world. Therefore, the succession of microbial communities in metallic mine soils over time is not clear. Compared with coal mines soils, the wastes soils of metallic mine contain higher sulfur, iron, and other metals, which are more likely to produce AMD, thereby causing serious environmental pollution. In this study, the “space for time” substitution was used to study changes of microbial communities over time in the dump of Nanshan Iron Mine in Anhui Province, China.

**Figure 1:**
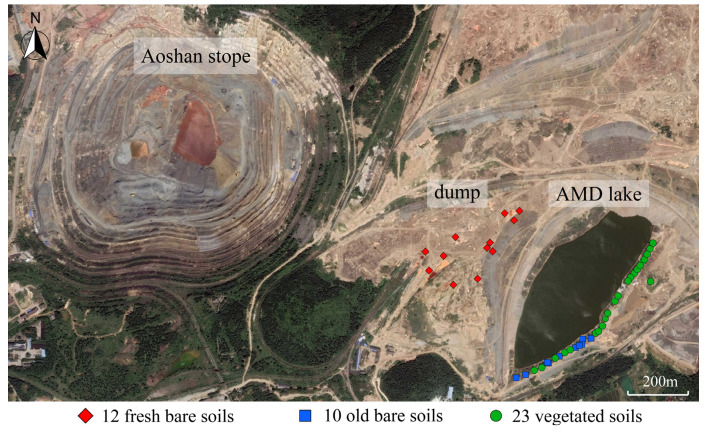
Sampling sites of three types of mine soils in the dump of Nanshan Iron Mine in Anhui Province, eastern China. Fresh bare soils stacked for less than 10 years were collected from the western side of the AMD lake, while the old bare soils and vegetated soils stacked for more than 40 years were sampled from the eastern side of the lake.

The Aoshan stope of Nanshan Iron Mine has been mined since the 1950s, and the main mineral component is pyrite. The stripped waste materials produced during the mining process were stacked in a nearby dumping yard (Figure 1). These high-iron-sulfur-containing mine wastes exposed to the surface were in contact with oxygen and water, forming a large amount of acid mine drainage under the oxidation of microorganisms. The acidic wastewater flowed down the mountain, polluting the local farmlands and drinking water sources. To control this pollution, Nanshan Iron Mine dug a large pit in the low-lying area at the center of the dump. The acid mine wastewater collected here, and an AMD lake formed in the 1970s (Figure 1). Since then, due to the obstruction by the AMD lake, the newly exfoliated materials were mainly lying on the western side of the lake. Therefore, the mine soils on the western embankment of the lake are relatively fresh, whereas those on the eastern side are relatively old, and most areas have been covered by vegetation.

The mine soils of different ages on the two sides of the AMD lakes offer an ideal opportunity to study the processes of microbial community succession in mine soils. We collected 45 soil samples from different areas of the dumpsite and studied the variation pattern of microbial communities using high-throughput sequencing. The study aimed to (i) compare the microbial communities of mine soils with different stacking times; (ii) characterize the major physicochemical parameters that control the microbial community and analyze the interplay between microorganisms and their habitats; and (iii) summarize the succession processes of microbial community structure and function from fresh mine soils to vegetated mine soils. Our results not only expand the current knowledge of acidophilic microbial community structure and function in response to physicochemical conditions in mine environments, but also contribute to the development of remediation strategies for mine soils.

## 2. Materials and Methods

### 2.1. Sampling Site Description and Sample Collection

Nanshan Iron Mine is located in the eastern part of Anhui Province, China (Figure 1) and is part of the Ningwu metallogenic belt in the middle and lower reaches of the Yangtze River. The region has a subtropical monsoon climate, with an average annual temperature of 16 °C and annual precipitation of about 1100 mm, reaching the lowest and highest daily average temperatures in January (3 °C) and July (28 °C) respectively.

The Aoshan stope, the major ore source of Nanshan Iron Mine, has been exploited for around 70 years and the mine wastes were disposed of in the nearby mine dump, which is about 1.8 km long and 1.7 km wide. Every year, the dump accumulated about 6.5–7.0 million tons of mine wastes. To study the succession process of the physicochemical properties and microbial communities of the mine soils in the dump, we collected three different types of soil samples in May 2017. Type 1 samples were collected from the western side of the lake and named F1–F12 (Figure 1 and Figure S1). The stacking time of these mine soil samples was less than 10 years, and there were several AMD ponds with different sizes around most of the sampling sites. The longest distance between the sampling points was about 353 m, and the sampling area was about 8.3 × 10^4^ m^2^. Type 2 samples, named O1–O10, were collected from the eastern side of the lake, where the soils have been stacked for over 40 years and were exposed on bare land. The longest distance between the sampling points of this type of sample was about 280 m. Type 3 samples were also collected from a portion of the eastern side. However, unlike Type 2 samples, these soils were from areas covered by vegetation, including herbs, shrubs, and trees (Figure S1). These samples were named V1–V23. Since vegetation covers most the eastern shore of the AMD lake, the longest distance between such sampling points was about 586 m.

About 500 g of each soil sample was collected, stored in an icebox, transported to the Geomicrobiology Laboratory of China University of Geosciences (Beijing) on the same day, and stored at −80 °C for subsequent analysis.

### 2.2. Determination of Physicochemical Parameters

The moisture content (MC) of the soil samples was determined by weighing the samples before and after drying them at 105 °C for 24 h. The dried soil samples were then used to determine other parameters. An organic carbon analyzer (Analytikjena, Jena, Germany) was used to determine total organic carbon (TOC), and an elemental analyzer (Elementar, Hanau, Germany) was employed to determine total nitrogen (TN) and sulfur (S). Soil samples were mixed with ultrapure water at a ratio of 1:10 (*w*/*v*) and placed on a stirrer to mix for 10 min. The mixture was left to stand for 30 min to dissolve the salts in the soils. The pH and electrical conductivity (EC) of the soil suspension were measured using a pH electrode (Thermo, Waltham, MA, USA) and a conductivity meter (Thermo, Waltham, MA, USA) respectively. The mixture of soil and water was centrifugated at 6000 rpm and the soluble metals in the supernatant were determined using ICP-AES (Thermo, Waltham, MA, USA), and anions were analyzed using ion chromatography (Thermo, Waltham, MA, USA). The soluble total phosphorus (TP) in the samples was determined by ammonium molybdate spectrophotometric method and ammonia nitrogen (NH_4_^+^-N) with Nessler’s reagent spectrophotometric method according to the water and wastewater monitoring and analysis methods [13].

### 2.3. DNA Extraction, PCR Amplification and High-Throughput Sequencing

Total DNA was extracted from 0.5 g soil samples using MPBio FastDNA Spin kit (MP Biomedicals, Santa Ana, CA, USA). High-throughput sequencing primer pairs F515 (5′-GTGCCAGCMGCCGGTAA-3′) and R806 (5′-GGACTACVSGGGGATCTAT-3′) were used to amplify the V4 hypervariable region of the 16S rRNA gene of both bacteria and archaea. The PCR reaction conditions were as follows: pre-denaturation at 94 °C for 3 min; denaturation at 94 °C for 30 s, annealing at 50 °C, extension at 72 °C for 60 s, a total of 35 cycles; and finally extended at 72 °C for 10 min.

The amplified PCR products were detected by agarose gel electrophoresis and purified using DNA Gel Recovery kit Ver.2.0 (Axygen, New York, NY, USA). The purified samples were sequenced by Ion Torrent (Thermo, Waltham, MA, USA) at the Chinese Academy of Agricultural Sciences.

### 2.4. Data Analysis

Mothur software [14] was used to analyze the high-throughput sequences. The “cluster” command was used to obtain operational taxonomical units (OTUs) with similarity greater than 97% and the “classify.seqs” command was employed to compare OTUs with silva.nr_v138 database to obtain the taxonomic classification of each OTU, with a confidence threshold of 80%, which was verified manually by BLAST. The “summary.single” command was used to calculate α-diversity indices including Shannon diversity index, Simpson diversity index, Chao1 richness index, and Heip evenness index, etc. By comparing the number of sequences assigned to a specific taxon with the total number of sequences obtained from the sample, the relative abundance of all OTUs in the sample was calculated. The physicochemical parameters and alpha diversity indices of three types of mine soil samples were compared using one-way analysis of variance (ANOVA).

The 300 most abundant OTUs were selected for the microbial network analysis using “iGraph” and “psych” to compare the differences in microbial interactions in different types of samples. The strong interactions with Spearman correlation coefficients of *ρ* > 0.6 and significance of *p* < 0.05 corrected by “BH” were shown in the network diagram. Each type of OTU was regarded as a network node. The color of nodes represents different phyla, node size is proportional to the link numbers (degree) of each node, and connection between nodes represents positive (red) and negative (blue) correlation. The main parameters describing the network topological characteristics used in this study included total links (the number of connections between nodes), negative links (the number of negative connections between nodes), positive links (the number of positive connections between nodes), connectance (the fraction of all possible links that are realized in a network), average degree (index of complexity of networks), average path length (the average network distance between all pairs of nodes), network diameter (the distance between the two most distant nodes), clustering coefficient (the average fraction of pairs of nodes connected to the same nodes that are also connected to each other, expressing the tendency of organisms to form cluster with relatively high-density ties), modularity (which demonstrates how well a network could be naturally divided into modules), centralization betweenness (which is close to 0 for a network where each node has the same betweenness, and the bigger the more difference among all betweenness values), and centralization degree (which is close to 1 for a network with star topology and in contrast close to 0 for a network where each node has the same degree). The network topological parameters have been previously described in detail by [15,16]. The microbial community stability of mine soils was evaluated by average variation degree (AVD), which reflects the variation degree of OTU relative abundance in each group. Lower AVD values indicate higher microbiome stability [17].

Statistical analyses were executed using various R packages (http://www.r-project.org/, accessed on 5 November 2021). Multiple statistical methods were employed to analyze microbial composition data of the three types of samples to ensure the reliability of results. Gplots, vegan, and heatmap.plus were used to cluster and visualize the relative abundance of the taxonomic units in different samples. Based on Bray-Curtis distance of OTUs, principal coordinate analysis (PCoA) and non-metric multidimensional scale analysis (NMDS) were implemented to compare the microbial communities of different types of samples. Pearson correlation coefficient was used to evaluate the relationship between diversity index and environmental factors. Mantel test was employed to analyze the relationship between microbial community composition and environmental factors, while “BioEnv” analysis was executed to find the combination of environmental factors which had the greatest influence on the composition of the microbial community. Canoco 5.0 software was used to calculate the influence of main environmental factors on the observed variations in microbial communities.

## 3. Results

### 3.1. Physicochemical Properties

Among the three types of samples, fresh bare soils on the western side of the AMD lake were the most acidic, with a pH range of 2.56–2.91 and the highest sulfur content of 2590–24,710 mg/kg (Table S1). The content of anions and cations in these soils was much higher than the other two types of samples, such as Fe (944.9–2356.5 mg/kg), Ca (471.6–8028 mg/kg), Mg (144.9–1714.2 mg/kg), and SO_4_^2−^ (1268.5–20,163.4 mg/kg). The high ionic concentration subsequently resulted in a high EC (870–4120 µS/cm). Due to the harsh physicochemical conditions of these soil samples, their content in TOC (1150–3590 mg/kg), TN (340–870 mg/kg), and TP (1.3–16.4 mg/kg) was low (Table S1).

The acidity of the old bare soil samples from the eastern side of the AMD lake was weaker than that of the fresh bare soils, with pH 2.93–3.33 (Table S1). Compared with the fresh bare soils, the ionic concentration and EC in the old bare soils were greatly reduced. Fe dropped to 317.1–1401.6 mg/kg, Ca to 68.1–2040.3 mg/kg, Mg to 12.7–819.6 mg/kg, and SO_4_^2−^ to 1070.1–6183.7 mg/kg, while EC reduced to 241–1605 µS/cm. On the contrary, the content of TOC and TN increased significantly to 2510–8940 mg/kg and 360–910 mg/kg, respectively (Tables S1 and S2). The TP of the samples was 2.3–8.3 mg/kg.

The vegetated soils samples on the eastern side of the AMD lake had the weakest acidity, with pH 3.48–4.64 (Table S1). Compared with the old bare soils, the ionic concentration and EC of these samples reduced further, with Fe (105.6–855.3 mg/kg), SO_4_^2−^ (192.1–1303.2 mg/kg), and EC (106–322 µS/cm). Moreover, the soils had the best nutrient conditions among the three types of soil samples with high TOC (1560–58,680 mg/kg), TN (390–3880 mg/kg), and TP (8.1–139.1 mg/kg) content (Tables S1 and S2).

### 3.2. Alpha Diversity and Microbial Community Composition in Three Types of Mine Soil Samples

**Figure 2:**
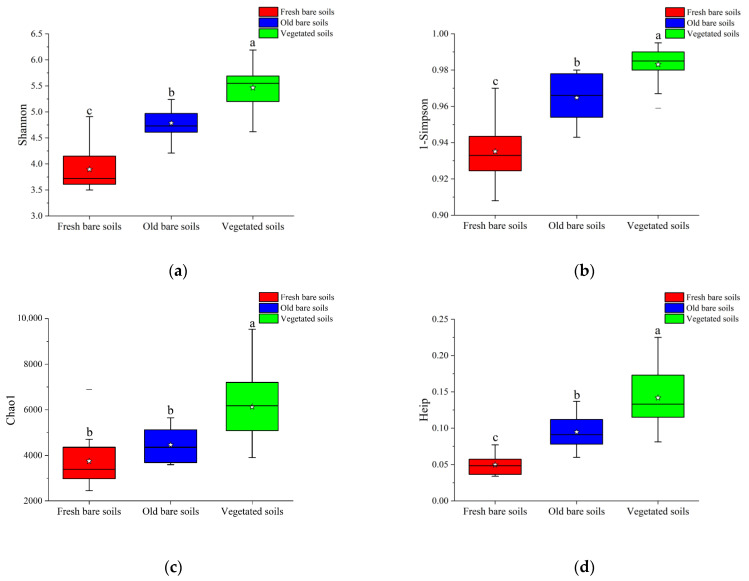
The alpha diversity indices Shannon (**a**), 1-Simpson (**b**), Chao1 (**c**), and Heip (**d**) of the three types of mine soil samples. Box plots display the first (25%) and third (75%) quartiles, the median, and the maximum and minimum observed values within each group. Data were analyzed by one-way ANOVA. Different lowercase letters above the bars indicate significant differences at *p* < 0.05 according to least significant difference (LSD) test.

After quality control of the high-throughput sequencing data, 11,305–83,628 high-quality sequences were obtained from each sample. To avoid bias due to difference in sequencing depths on subsequent analyses, we unified the sequencing depth of all samples to 11,000. These sequences were clustered with 97% similarity, and a total of 175,870 OTUs were obtained. Among them, sample F3 in fresh bare soils had the least number of OTUs (708), whereas sample V23 from the vegetated soils had the largest number of OTUs (2384) (Table S3). All alpha diversity indices including Shannon, Simpson, Chao1, and Heip increased gradually from fresh bare soils and old bare soils to vegetated soils (Figure 2), and the diversity indices of vegetated soils were significantly higher than those of the other two types of samples (Table S4), suggesting their remarkedly higher biodiversity.

A total of 35 bacterial and six archaeal phyla were obtained through taxonomic analysis, with bacteria and archaea accounting for 86.4% and 13.6% of all sequences respectively. Bacteria were dominant in all samples, with average abundances of 73.1% (fresh bare), 81.2% (old bare), and 97.9% (vegetated soils), whereas the proportion of archaea in the three types of samples decreased gradually.

**Figure 3:**
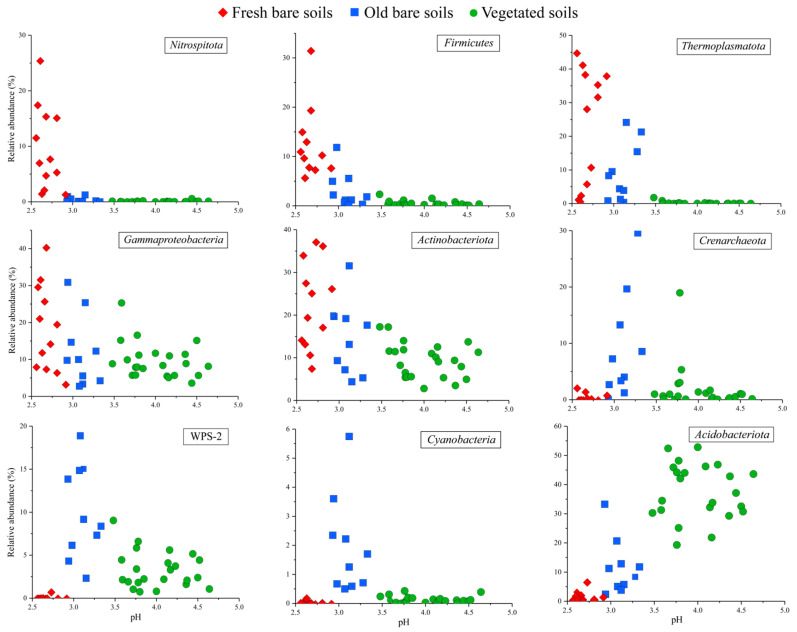
The relative abundance of major phyla/classes along a pH gradient in the three types of mine soil samples.

*Thermoplasmatota* and *Actinobacteriota* were the dominant phyla in the most acidic fresh bare soils, accounting for 26.3 ± 16.1% and 20.2 ± 9.3%, respectively (Figure 3 and Figure S2). The proportion of *Thermoplasmatota* decreased in old bare soils (9.1 ± 8.7%) and vegetated soils (0.2 ± 0.4%). The distribution of *Actinobacteriota* was consistent with that of *Thermoplasmatota*, and its abundance in the old bare soils and vegetated soils decreased to 13.3 ± 6.5% and 9.4 ± 3.9%, respectively. Similarly, *Gammaproteobacteria* (17.8 ± 12.6%), *Firmicutes* (12.4 ± 7.1%), and *Nitrospirota* (10.3 ± 7.8%), which had higher abundance in fresh bare soils, were also significantly reduced in the other two types of samples. WPS-2 (10.1 ± 6.3%) and *Crenarchaeota* (10.8 ± 10.2%) were the dominant phyla in the old bare soils. In addition, there was a proportion of *Cyanobacteria* (1.7 ± 0.1%) in these samples, while its average abundance in the other two types of samples was less than 0.1%. Unlike the first two types of samples, *Acidobacteriota* occupied a dominant position (39.1 ± 9.6%) in the least acidic vegetated soils, while the average abundance of all other phyla was less than 10% (Figure 3 and Figure S2).

**Figure 4:**
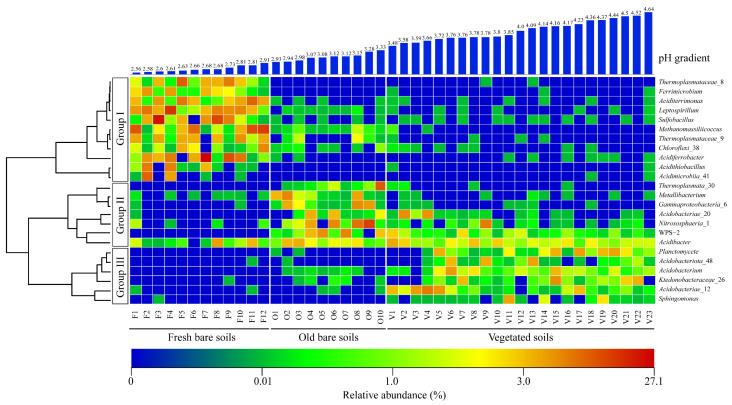
The percentage of the abundant microbial genera as revealed by the 16S rRNA gene in the mine soils. The color scale quantifies the relative abundance percentages from low (blue) to high (red).

Relative abundances and distributions of bacterial and archaeal communities at genus level across the soil samples were visualized in a heatmap (Figure 4). The major genera in different types of soils formed three distinct groups (I, II, and III) along a pH gradient (Figure 4). Group I mainly consisted of autotrophic iron-sulfur oxidizing bacterial genera from fresh bare soils, such as *Acidiferrobacter* (7.3 ± 8.4%), *Leptospirillum* (7.1 ± 5.8%), and *Sulfobacillus* (6.2 ± 7.9%) (Figure 4). However, in the old bare and vegetated samples, their abundances dropped sharply or were not detectable.

Most of the sequences in the old bare soil samples were not closely related to any of the cultivated strains, and only a few sequences could be classified at genus level clustered in Group II (Figure 4), such as heterotrophic bacteria *Acidibacter* (2.6 ± 1.3%) and *Metallibacterium* (2.3 ± 3.3%).

The genera identified from the vegetated soil samples were mainly from the phylum *Acidobacteriota*, such as *Acidobacterium*, *Acidipila*, *Granulicella*, and *Acidicapsa*, which clustered in Group III (Figure 4).

### 3.3. Microbial Co-Occurrence Network and Community Stability of the Mine Soils

**Figure 5:**
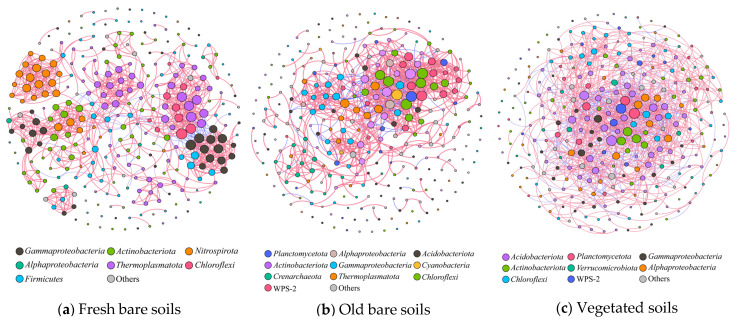
Microbial co-occurrence networks in the three types of mine soil samples. The size of nodes is proportional to the link numbers (degree) of each node. The links between each pair of nodes represent positive (in pink) and negative (in blue) interactions with Spearman correlation *ρ* > 0.6 and *p* < 0.05.

The microbial network analysis was performed using the main OTUs selected from the three types of soil samples with a strong correlation of Spearman *ρ* > 0.6 and *p* < 0.05 (Figure 5). The results showed that the total links of vegetated samples were much higher than those of the other two types of samples (Table S5). In all three types of samples, the number of positive correlations was higher than the number of negative correlations, but the proportion of positive correlations decreased gradually from fresh bare soils (91.5%) and old bare soils (86.0%) to vegetated soils (68.8%).

Similar to total links, parameters such as connectance and average degree gradually increased in the three types of samples, indicating that the microbial networks in the mine soil samples became more complex over time while parameters such as average path length and network diameter gradually decreased (Table S5), showing the relationship among microbes became closer and their interaction got stronger from fresh bare soils and old bare soils to vegetated soils.

The average variation degree (AVD) of microbiome in vegetated soils was obviously lower than those of the other two types of samples (Table S6), indicating the microbial communities in vegetated soils were more stable and had a higher system resistance to environmental change compared with the communities in bare soils, which is consistent with the relatively high proportion of negative correlations (31.2%) among microbes in vegetated soils, indicative of community stability.

### 3.4. Effects of Environmental Factors on the Microbial Diversity and Community Composition in the Mine Soils

Pearson correlation analysis showed that pH and EC had the strongest correlation with Shannon and 1-Simpson indexes. The pH had a significant positive correlation with Shannon index and 1-Simpson index (r = 0.856, *p* < 0.01; r = 0.781, *p* < 0.01), respectively, whereas EC was negatively correlated with both indices (r = −0.830, *p* < 0.01; r = −0.849, *p* < 0.01), indicating that the diversity of microbial community was enhanced with the increase in pH and the decrease in EC (Figure S3).

**Figure 6:**
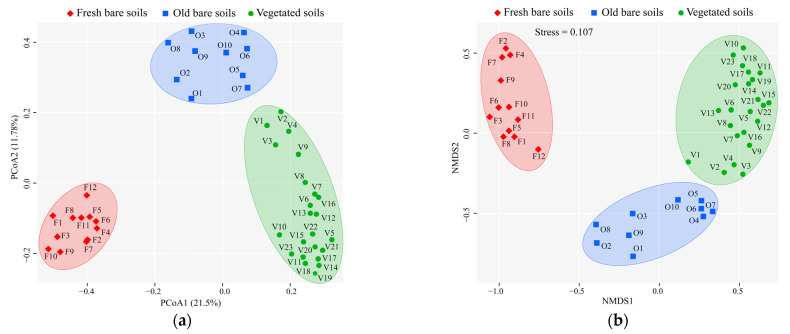
Principal coordinate analysis (PCoA) (**a**) and non-metric multidimensional scaling (NMDS) (**b**) of microbial community composition in the studied mine samples. Both analyses showed that the 45 mine soils clustered into three distinct groups based on sample type.

ANOSIM confirmed that there were significant differences in microbial composition among three types of samples (r = 0.941, *p* = 0.001). The PCoA and NMDS results showed that 12 fresh bare, 10 old bare, and 23 vegetated soil samples were clustered into different groups, respectively (Figure 6). Mantel test indicated that the microbial community composition in the mine soils had a strong correlation with environmental factors (r = 0.704, *p* < 0.001). The BioEnv analysis revealed that the combination of pH and EC had the greatest impact on the composition of the microbial community with the highest correlation (r = 0.736) (Table S7). Furthermore, CCA also emphasized that EC and pH were the most influential environmental factors with the highest degree of explanation (7.2% and 6.7%) respectively (Table S8).

## 4. Discussion

### 4.1. Succession of the Physicochemical Properties in the Mine Soils

The long history of the dumpsite at the Aoshan stope and the barrier effect of the AMD lake makes the site suitable for studying the succession process of physicochemical properties and microbial communities of mine soils.

The mine soils on the western side of the AMD lake were relatively fresh because of its proximity to the quarry. The fresh mine soils piled in the open contained a large amount of reduced iron and sulfur, producing a large amount of sulfuric acid and dissolved metal ions through vigorous oxidation reaction. Therefore, these soil samples were the most acidic, had the highest ion concentration and electrical conductivity, and the environmental conditions were the most extreme. This is consistent with previous research reports from metallic mine dumps [18].

On the contrary, the mine soils on the eastern side of the lake have a long stacking history, and the reduced iron and sulfur in these soils have nearly been exhausted. Compared with the fresh soils, the acidity of the old bare mine soils, although exposed on the surface, has been significantly weakened. The concentrations of various metal ions and electrical conductivity has also reduced, thereby mitigating the severity of the environmental conditions. Similarly, the concentrations of TOC and TN in this type of mine soil samples were much higher than those in fresh bare soil, which proves that various nutrients have gradually accumulated in this type of sample, and the soils were becoming ready for pioneer plant colonization.

The acidity and ionic concentration of the vegetated soil samples weakened further, making them suitable for the growth of plants such as bermudagrass, reeds, thorny shrubs, and eucalyptus trees (Figure S1). The growth of plants in turn improved soil quality dramatically. Therefore, the nutritional conditions of the vegetated soils were the best among the three types of sampled soils. Similar to our results, a study by Gagnon et al. on the abandoned gold mine tailings in Quebec, Canada also found that the organic matter content of tailings covered with vegetation was much higher than that without vegetation cover, and the higher the vegetation density, the higher the organic matter content of the tailings [19].

### 4.2. Succession of Microbial Communities in Different Mine Soils

Since the three types of mine soil samples were in different succession stages, their physicochemical characteristics were relatively discriminative. Correspondingly, the microbial communities of the three types of mine soil samples varied remarkably. Among the physicochemical factors, pH and EC had the most important impact on the microbial community composition. Usually, soil pH affects the chemical form, concentration, and availability of ions considerably. Therefore, pH should be the most significant driver of microbial diversity and community structure in the three types of mine soil samples, which is consistent with previous studies in normal soil systems [20,21].

The fresh bare soil samples on the western side of the AMD lake contained a large amount of reduced iron and sulfur, hence the abundance of iron-sulfur oxidizing bacteria, such as *Acidiferrobacter, Leptospirillum, Sulfobacillus, Acidithiobacillus*, and *Ferrimicrobium*. All of these genera are commonly detectable in waste soils from metallic mines. *Acidiferrobacter* is a facultatively anaerobic and thermotolerant genus with optimal pH about 2.0 that grows chemoautotrophically through oxidizing ferrous, elemental sulfur, sulfide, and tetrathionate and uses molecular oxygen and ferric iron as electron acceptors [22]. *Leptospirillum* has a pH optimum of 1.3–2.0, and uses ferrous iron as the only electron donor and oxygen as the electron acceptor to obtain energy [23]. *Sulfobacillus*, a genus commonly found in extremely acidic and metal-rich environments with optimal pH 1.9–2.4, can oxidize Fe^2+^, S^0^, S_2_O_3_^2−^, S_4_O_6_^2−^, and sulfide minerals, playing an important role in the formation of AMD and biometallurgical processes [24]. The iron-sulfur oxidizers accelerated the oxidation of sulfide in the waste soils and generated toxic AMD. Under the inhibition of extremely low pH and high ion concentration, the microbial diversity and evenness of these samples were the lowest and the microbial network was the simplest. The low biodiversity and network complexity resulted in the low stability of the microbial community in the fresh bare soils [25,26], which is consistent with the results of the community stability index AVD. Usually, increasing stress in the environment could destabilize microbiomes and undermine their ecosystem services [27]. In the acidic and oligotrophic mine soil environments, microbes can survive the harsh environment through synergistic interactions, reflected by the highest proportion of positive correlations in the microbial network of fresh bare soils.

The iron and sulfur in the old bare soils on the eastern side of the lake have been hugely oxidized because of their long stacking history. Therefore, the iron-sulfur oxidizers abundant in the fresh soils were largely absent in the old bare soils. In contrast, some microorganisms that can increase environmental pH through metabolism such as *Acidibacter* and *Metallibacterium* began to appear. *Acidibacter* can catalyze the reductive dissolution of the ferric iron mineral schwertmannite, causing the environmental pH to increase [28]. Likewise, *Metallibacterium* can produce ammonia by metabolizing casein, thereby increasing the pH of its microenvironment. This is why its optimal pH is 5.5 but it can survive in environments of pH 2.5 [29]. Through the influence of these microorganisms, the acidity of the old bare soils was weakened dramatically.

The old bare soils also had an abundance of WPS-2 (10.1 ± 6.3%). This bacterial candidate phylum has been detected in various environments around the world and is particularly abundant in extreme environments, such as cold, acidic, and organic carbon deficient environments. Many members of this phylum have photosynthetic pigments and can perform anoxygenic photosynthesis [30,31]. It does not compete with the traditional photosynthetic *Cyanobacteria*, because their absorption spectra are complementary [32]. It has been reported that WPS-2 tends to thrive in bare environments rather than those covered by vegetation [33]. This may be because this bacterium needs to obtain energy from sunlight. The preference was also confirmed in this study. Moreover, some photoautotrophic WPS-2 species can also carry out chemoautotrophic biosynthesis through oxidizing rare gases such as H_2_ and CO and was assumed as the main primary producer in some harsh environments [30,34]. Phylogenetic analysis showed that 38% of the WPS-2 in this study clustered with photo/chemo-autotrophic members (Figure S4).

In addition to WPS-2, *Cyanobacteria* also accounted for a considerable abundance in old bare soils compared with the other types of samples (Figure 3). It is generally believed that such microorganisms are mainly distributed in aquatic environments, but in fact, *Cyanobacteria* is an important primary producer in many extreme terrestrial environments such as deserts, steppes, and high-elevation soils [35]. Due to the abundance of photo and chemoautotrophic bacteria, TOC accumulated gradually in the old bare soils and became remarkably higher than that in the fresh bare samples. Lower acidity, ion concentration, and better nutritional conditions in the old bare soils significantly improved the microbial diversity and evenness and the interactions among microbes also became complex.

Compared with the other two types of samples, vegetated soils had the most favorable environmental conditions. Accordingly, microbial diversity and evenness were at their peak, and the interactions among microorganisms were more complex, which can promote resistance of microbial community to external interference, hence contributing to a stable ecological function.

The abundance of *Acidobacteriota* increased across the three types of samples, up to 39.1 ± 9.6% in the vegetated soils, becoming the predominant group. Similar to our study, an increasing abundance of *Acidobacteriota* with increasing restoration ages has also been reported in other research focusing on post-mined chronosequence soils [36]. *Acidobacteriota* is widely distributed in various land ecosystems around the world and is particularly abundant in soils [1,37]. However, *Acidobacteriota* is difficult to isolate under laboratory conditions and, according to the 16S rRNA gene sequence, it has been divided into 26 subdivisions. Currently, only seven subdivisions (namely the subgroups 1, 3, 4, 6, 8, 10, and 23) are represented by taxonomically described members, most of which are acidophilic, aerobic, chemo-heterotrophic, and prefer to grow in oligotrophic environments [38,39,40,41,42].

Acidobacterial species play important ecological functions in the soil environment. Firstly, they can promote the circulation of elements in the soil system. *Acidobacteriota* can decompose complex carbohydrate polymers from plants and fungi in the soil, such as cellulose, hemicellulose, chitin, xylan, and lignin [43]. Secondly, they can secrete extracellular polymers, exopolysaccharides (EPS) [44], and promote the formation of soil structure and aggregates [45], and accumulation of water and nutrients, thereby changing the microenvironment of the plant rhizosphere and establishing plant-microbe interactions [46]. It was found that three strains of *Acidobacteriota* can produce EPS, allowing the strains to adhere to the roots of *Arabidopsis thaliana* to form a biofilm, which promotes the absorption of water and nutrients by plants. Thirdly, some strains of *Acidobacteriota* can produce auxin (indole-3-acetic acid, IAA) and siderophore to promote plant growth directly [47]. Therefore, *Acidobacteriota* plays a great role in the improvement of environmental conditions and ecological restoration of the mine soils. Its dominance indicates the soil ecosystem has become mature. Considering the significant role played by Acidobacterial species in the natural reclamation process of mine soils, we need to either develop favorable conditions or inoculate Acidobacterial strains into the mine soils to accelerate the artificial restoration process of mine dumps.

## 5. Conclusions

Among the three types of mine soil samples from the dump of the Aoshan stope, the fresh bare samples had the lowest pH, highest ion concentrations, and were the most deficient in nutrients while the acidity and ion concentration of old bare samples decreased significantly, and the nutritional conditions improved. Vegetated samples had the lowest acidity, lowest ion concentration, and the highest nutrient concentrations. In the fresh mine soils, the iron/sulfur-oxidizers such as *Acidiferrobacter, Leptospirillum*, and *Sulfobacillus* were dominant, resulting in the strong acidity. Bacteria from genera *Acidibacter*, *Metallibacterium*, and phyla *Cyanobacteria*, WPS-2 were abundant in the old bare samples, which contributed to the pH increase and TOC accumulation in the soils, respectively. *Acidobacteriota* predominated in the vegetated samples, which promoted soil material circulation, nutrient accumulation, and plant growth significantly. The microbial diversity and evenness of the three types of soils increased gradually from fresh bare and old bare to vegetated soils, with more complex microbial networks, suggesting that the microbial community became more mature with time and microorganisms co-evolved with the mine soils. The pH was the most important driver of microbial community variation in the mine soils. The results of this study provided scientific information for the ecological restoration of mining areas. Since Acidobacterial species played a significant role in the natural reclamation process of mine soils, it is suggested to develop favorable conditions or inoculate Acidobacterial strains into the mine soils in the artificial restoration of mine dumps.

## Data Availability

Raw data were deposited in SRA database under BioProject PRJNA759289.

## Supplementary Materials

The following are available online at https://www.mdpi.com/article/10.3390/microorganisms9122463/s1, Table S1: Physicochemical parameters of the mine soils, Table S2: The analysis of variance (ANOVA) of main physicochemical parameters of the three types of mine soil samples, Table S3: The alpha diversity indices of the mine soil samples, Table S4: The analysis of variance (ANOVA) of main alpha diversity indices of the three types of mine soil samples, Table S5: Topological parameters of the microbial networks in the three types of mine soil samples, Table S6: The average variation degree (AVD) of different samples and groups, Table S7: The impact of combinations of physicochemical parameters on the microbial community composition in the mine soils, Table S8: Canonical correspondence analysis (CCA) of microbial community composition data and main physicochemical parameters, Figure S1: A catalog of selected photos from mine soils sampling sites in the dump of the Nanshan Iron Mine in Anhui Province, China, Figure S2: The relative abundance of major phyla/classes along a pH gradient in the 3 types of mine soil samples, Figure S3: Correlation relationship between physicochemical parameters and diversity indices, pH with (a) Shannon and (b) 1-Simpson, EC with (c) Shannon and (d) 1-Simpson, Figure S4: Phylogenetic tree based on 16S rRNA gene sequences of WPS-2 from metagenomic assembled genome and the mine soils. The 16S rRNA gene sequences obtained in this study are in bold characters, 38% of which were clustered with Ca. Hemerobacter limicola, a photo/chemo-autotrophic species.
