# Supplementary material for: Succession of Microbial Communities in Waste Soils of an Iron Mine in Eastern China
Source: Microorganisms. 2021 Nov 29;9(12):2463. doi: 10.3390/microorganisms9122463 (PMC8704403; doi:10.3390/microorganisms9122463)
Supplement: Supplementary file 1 [file microorganisms-09-02463-s001.zip › microorganisms-1417376-supplementary.pdf]

Table S1. Physicochemical parameters of the mine soils

Sample	pH	MC %	Al mg/kg	Ca mg/kg	Cu mg/kg	Fe mg/kg	K mg/kg	Mg mg/kg	Mn mg/kg	Na mg/kg	Cl^- mg/kg	SO_4^{2-} mg/kg	TN mg/kg	TOC mg/kg	EC uS/cm	S mg/kg	TP mg/kg	NH_4^+-N mg/kg
F1	2.56	16.8	1279	3376	4.6	1387.4	22.6	418	61.1	250.4	330.4	5230.1	740	3240	1490	14552	1.3	7.27
F2	2.58	12.0	979.2	1062.3	24.5	1149.6	218.1	617.1	59.2	310.8	217.2	1268.5	590	2970	3830	24710	11.2	1.57
F3	2.60	13.3	1613.1	6366	50.3	2356.5	87.9	1714.2	137.2	311.4	40.7	20163.4	370	3310	3310	18040	18.3	2.12
F4	2.61	15.9	763.8	471.6	14.5	1041.3	119.4	292.5	31.4	354.6	41.1	2906.1	650	3590	4120	15520	7.1	2.71
F5	2.63	12.7	927.2	1363.5	7.1	1298.3	118.6	155.8	9.1	382.5	32.7	8304.1	400	1930	870	3689	14.5	4.1
F6	2.66	14.1	969.6	8028	14.8	1036.2	233.1	634.2	38	247.8	323.3	12709.7	400	1600	2850	17020	16.4	1.69
F7	2.68	7.9	232.9	2030.7	4.2	1099.2	283.5	200.6	12.6	357.9	49	4293.1	340	1930	2910	10530	3.7	0.4
F8	2.68	17.7	1313	3703	15.5	1015	37.8	326.7	75.6	240.4	55.8	5803.0	870	1150	3440	6591	2.8	4.16
F9	2.73	16.5	422.1	6234	4.5	1142.4	198.6	392.1	45.8	498.9	129.7	11318.2	350	1690	3100	2590	8.7	2.48
F10	2.81	8.2	145.6	4626	1.9	1381.5	92.7	141.9	9.9	368.1	51.4	8935.6	350	2570	2930	5610	9.2	1.46
F11	2.81	13.1	1404	2230	6.1	944.9	23.3	287.9	41.3	349.8	26.4	6460.4	350	3140	1930	15108	9.8	7.56
F12	2.91	13.4	258.5	1536.3	1.7	1212.2	77.2	274.6	16.6	351.4	129.4	5190.1	480	2120	1600	14614	6.8	8.06
O1	2.93	19.1	1394.4	981	30.6	1401.6	290.1	819.6	152.7	484.6	272.8	5956.3	910	8320	1605	10520	7.1	9.49
O2	2.94	14.2	613.5	904.9	14.9	1094.6	270.8	817.2	66.4	701.3	99.2	6183.7	365	3460	685	5170	4.3	7.58
O3	2.98	15.0	475.5	510.6	4.8	1286.7	138.9	64.8	15.0	398.1	356.2	2875.1	600	3480	241	9060	2.3	4.78
O4	3.07	22.4	906.9	318	9.7	1094.1	310.8	12.7	78.2	313.9	249.3	5226.7	560	7130	485	5711	3.9	12.76
O5	3.08	14.0	1130	565.2	1.9	317.1	231	69.6	33.9	120.6	333.2	1202.4	610	5810	377	6171	6.9	6.82
O6	3.12	15.4	596.7	68.1	5.1	1262.7	226.8	122.6	14.9	403.8	411.8	1632.3	440	3380	385	4410	3.5	4.7
O7	3.12	13.3	929.1	323.4	9.6	953.7	106.7	13.7	94.4	216.4	571.9	3972.9	840	8940	320	3464	6.4	15.42
O8	3.15	23.1	1299.3	2040.3	33.2	1291.8	196.3	803.4	37.0	591.5	124.6	3046.7	836	3680	285	6931	8.3	6.43
O9	3.28	21.3	684.6	903.6	7.9	759.3	295.5	118.9	10.7	404.4	286.3	2962.3	370	3260	579	6810	6.2	0.22
O10	3.33	14.5	886.5	554.4	9.3	853.2	20.4	107.9	17.9	594.6	322.0	1070.1	360	2510	294	3840	8.1	12.21
V1	3.48	15.8	923.1	494.1	6.6	855.3	338.1	195.8	17.5	524.4	17.8	906.3	390	4335	322	5980	8.1	6.24
V2	3.58	16.2	589.6	895.2	4.5	452.3	137.7	179.8	37.2	320.6	33.2	794.9	470	1560	199	2147	23.8	2.91

MC: moisture content

Table S1 (continued)

Sample	pH	MC %	Al mg/kg	Ca mg/kg	Cu mg/kg	Fe mg/kg	K mg/kg	Mg mg/kg	Mn mg/kg	Na mg/kg	Cl^- mg/kg	SO_4^{2-} mg/kg	TN mg/kg	TOC mg/kg	EC uS/cm	S mg/kg	TP mg/kg	NH_4^+-N mg/kg
V3	3.59	16.6	1712	545.9	3.1	599.7	198.5	95.6	39.7	368.7	133.1	398.4	460	2030	279	1201	67.7	8.06
V4	3.66	12.3	793.2	799.2	11.5	679.5	101.1	107.8	68.6	220.8	137.9	1303.2	1420	4740	286	3755	43.7	5.94
V5	3.72	11.9	921.3	1431.3	5.8	446.8	91.8	231.6	9.3	475.6	72.7	295.6	860	6830	319	5487	13.8	7.67
V6	3.76	9.9	1083.6	902.4	7.8	600	591	175.0	108.9	403.5	145.1	1276.1	1250	15750	283	2330	21.6	3.06
V7	3.76	13.0	299.7	517.5	6.7	595.6	623.7	371.6	20.7	129.3	53.6	192.1	830	6190	278	1538	12.1	4.38
V8	3.78	15.5	983.7	513.3	14.3	682.8	562.8	204.8	43.4	613.8	19.2	425.5	480	7075	189	6100	48.9	2.76
V9	3.78	17.4	1230.9	600.9	9.4	632.4	155.1	161.9	23.2	537	258.9	547.4	880	7110	263	5594	23.2	8.61
V10	3.80	22.6	397.7	1392.9	2.3	603.4	228.8	88.6	23.8	334.2	33.1	388.3	3880	58680	177	4152	14.5	3.87
V11	3.85	17.7	96.41	380.6	4.5	105.6	82.5	151.2	41.1	139.8	41.6	216.1	1480	14580	293	2242	23.3	3.03
V12	4.00	22.6	837.9	946.5	10.2	578.1	362.1	188.8	39.1	483	207.8	280.1	2190	33900	187	5560	66.9	3.81
V13	4.09	12.4	1223.7	1424.4	17.2	555.3	252	290.1	96.6	726.3	29.1	599.1	690	12400	259	1516	46.4	9.97
V14	4.14	12.7	1591	841.8	3.3	455.2	525.7	96.4	40.7	120.8	55.9	363.1	1280	10570	110	2263	32.5	8.12
V15	4.16	8.4	1638.9	837.6	7.1	503.1	272.7	359.9	21.0	315.9	44.6	616.3	570	8470	126	2492	25.3	10.32
V16	4.17	14.2	446	688.8	3.8	414.0	265.2	156.3	41.4	280.4	33.2	290.6	1000	9890	170	1245	41.3	3.31
V17	4.23	13.7	956.1	891.6	7.0	604.8	194.1	219.7	57.7	301.5	207.8	221.9	700	8860	106	3030	119.5	1.23
V18	4.36	14.2	732.3	1831.8	10.3	525.9	168.0	246.9	103.3	330	348.7	386.9	1810	13000	108	4030	57.6	1.06
V19	4.37	15.7	1029	1553.4	16.1	590.7	181.4	275.6	69.6	470.1	42.8	216.9	820	9350	114	2300	139.1	1.87
V20	4.44	12.9	1072.2	808.2	18.6	468	230.7	266.5	167.5	334.5	6.5	253.5	490	5835	190	2300	71.1	9.95
V21	4.50	14.9	1305.6	779.1	15.5	490.8	438	273.5	741.6	348.1	259.2	588.6	760	7530	108	2607	63.4	5.19
V22	4.52	9.4	1125.3	1593.3	18.4	469.5	389.1	106.2	82.0	424.3	23.1	201.3	1130	7040	112	1596	48.7	5.24
V23	4.64	10.4	803.7	2030.7	4.8	523.2	401.1	522	169.4	309.9	20.9	225.8	1850	17500	118	1270	45.3	4.54

MC: moisture content

Table S2. The analysis of variance (ANOVA) of main physicochemical parameters of the three types of mine soil samples

Group	pH	SO_4^{2-} (mg/kg)	TN (mg/kg)	TOC (mg/kg)
Fresh bare soils	2.69±0.11c	7715.19±5124.82c	490.83±179.52b	2436.67±801.24b
Old bare soils	3.1±0.13b	3412.85±1883.31b	589.10±211.14b	4997.00±2357.39b
Vegetated soils	4.02±0.34a	477.74±321.47a	1116.96±778.64a	11879.35±12168.07a

Different lowercase letters within a column indicate significant difference at $P < 0.05$ according to LSD test. Values are mean±standard deviation.

Table S3. The alpha diversity indices of the mine soil samples

Sample	Goods coverage	OTUs	Shannon	Simpson	Chao1	ACE	Heip
F1	0.92	1259	4.44	0.038	4611.56	8998.24	0.066
F2	0.93	1090	3.64	0.078	3349.08	5086.20	0.034
F3	0.95	708	3.58	0.068	2461.51	4089.44	0.049
F4	0.93	1025	3.64	0.082	3701.24	6193.29	0.036
F5	0.92	1243	4.30	0.052	4700.69	9743.68	0.058
F6	0.88	1748	4.91	0.030	6891.32	11284.97	0.077
F7	0.94	921	3.51	0.092	2922.05	4544.60	0.035
F8	0.95	766	3.80	0.073	2449.69	3934.50	0.057
F9	0.94	875	3.50	0.072	3424.10	6362.59	0.037
F10	0.94	840	3.68	0.066	3324.19	6177.61	0.046
F11	0.94	881	3.76	0.066	3031.84	5381.37	0.048
F12	0.94	987	4.00	0.061	4094.35	6751.87	0.054
O1	0.93	1107	4.21	0.053	3637.19	6400.14	0.060
O2	0.86	1369	5.24	0.020	5647.12	11446.25	0.137
O3	0.92	1348	4.97	0.022	4790.51	8133.48	0.106
O4	0.93	1165	4.97	0.020	3592.80	6103.49	0.123
O5	0.90	1593	5.19	0.022	5478.50	9184.40	0.112
O6	0.92	1219	4.69	0.037	4457.58	7265.75	0.088
O7	0.93	1185	4.72	0.031	3684.38	6001.69	0.094
O8	0.93	1135	4.52	0.044	3893.72	6989.34	0.080
O9	0.90	1648	4.74	0.057	5119.56	8206.22	0.069
O10	0.92	1275	4.61	0.046	4254.45	7027.55	0.078
V1	0.89	1664	5.36	0.016	6308.81	11109.21	0.127
V2	0.91	1398	5.08	0.020	5698.63	10112.74	0.115
V3	0.92	1266	4.87	0.028	4054.66	7237.83	0.102
V4	0.92	1250	4.62	0.041	4051.30	6931.24	0.081
V5	0.91	1455	5.12	0.023	5076.53	9105.32	0.114
V6	0.86	2133	5.57	0.016	8635.71	16931.21	0.123
V7	0.89	1868	5.85	0.009	5870.44	10541.10	0.186
V8	0.89	1702	5.25	0.019	6399.48	11571.16	0.111
V9	0.92	1350	4.97	0.033	3905.00	6829.44	0.106
V10	0.88	1871	5.58	0.013	6174.38	11116.68	0.142
V11	0.90	1632	5.28	0.019	5416.26	9486.32	0.120
V12	0.91	1481	5.55	0.010	5502.41	8910.93	0.173
V13	0.85	2101	5.79	0.014	8114.09	16097.43	0.155
V14	0.89	1811	5.80	0.008	6283.11	10370.65	0.182
V15	0.90	1551	5.20	0.022	5429.35	8980.23	0.116
V16	0.89	1761	5.53	0.014	6330.39	10696.50	0.143
V17	0.85	2221	5.69	0.015	9542.58	16343.68	0.133
V18	0.87	2172	6.19	0.005	6435.50	11041.46	0.225
V19	0.88	1912	5.67	0.011	7444.71	13439.93	0.152
V20	0.87	2018	5.52	0.017	7198.40	14240.77	0.124
V21	0.92	1444	5.57	0.010	4386.88	6856.95	0.181
V22	0.91	1582	5.65	0.010	4740.61	7413.20	0.179
V23	0.85	2384	6.02	0.014	7987.55	15191.31	0.173

Table S4. The analysis of variance (ANOVA) of main alpha diversity indices of the three types of mine soil samples

Group	Shannon	Simpson	Chao1	Heip
Fresh bare soils	3.9±0.44c	0.065±0.018c	3746.80±1177.40b	0.05±0.013c
Old bare soils	4.79±0.31b	0.035±0.014b	4455.58±733.77b	0.095±0.025b
Vegetated soils	5.47±0.38a	0.017±0.008a	6129.86±1470.73a	0.142±0.035a

Different lowercase letters within a column indicate significant difference at $P < 0.05$ according to LSD test. Values are mean±standard deviation.

Table S5. Topological parameters of the microbial networks in the three types of mine

soil samples

Parameters	Fresh bare soils	Old bare soils	Vegetated soils
Total link numbers	904	829	1252
Positive link numbers (percent)	827 (91.5%)	713 (86.0%)	862 (68.8%)
Negative link numbers (percent)	77 (8.5%)	116 (14.0%)	390 (31.2%)
Connectance	0.027	0.032	0.034
Average path length	7.411	4.785	3.647
Average degree	7.008	7.272	9.206
Modularity	0.751	0.546	0.446
Network diameter	20	14	10
Clustering coefficient	0.618	0.476	0.364
Centralization betweenness	0.311	0.189	0.076
Centralization degree	0.066	0.096	0.121

Table S6. The average variation degree (AVD) of different samples and groups

Sample	Sample AVD	Group AVD
F1	0.6034	
F2	0.6689	
F3	0.6012	
F4	0.6713	
F5	0.7935	0.6869
F6	0.7533	
F7	0.7559	
F8	0.6614	
F9	0.6779	
F10	0.5941	
F11	0.7031	
F12	0.7589	
O1	0.7829	
O2	0.7892	
O3	0.6027	
O4	0.6324	
O5	0.7153	0.6928
O6	0.6306	
O7	0.7644	
O8	0.6834	
O9	0.6853	
O10	0.6419	
V1	0.7126	
V2	0.6846	
V3	0.7064	
V4	0.6120	
V5	0.5543	
V6	0.6244	
V7	0.6284	
V8	0.5742	
V9	0.5474	0.6282
V10	0.6707	
V11	0.6548	
V12	0.5975	
V13	0.5832	
V14	0.6168	
V15	0.6758	
V16	0.6266	
V17	0.5969	
V18	0.7133	
V19	0.5562	
V20	0.5966	
V21	0.5573	
V22	0.6628	
V23	0.6948	

Table S7. The impact of combinations of physicochemical parameters on the

microbial community composition in the mine soils

Physicochemical parameters	Size	Correlation
pH	1	0.629
pH EC	2	0.736
pH SO_4^{2-} EC	3	0.687
pH SO_4^{2-} EC S	4	0.667
pH Cl^- SO_4^{2-} EC S	5	0.648
pH MC Cl^- SO_4^{2-} EC S	6	0.625
pH MC Ca Cl^- SO_4^{2-} EC S	7	0.611
pH MC Ca Cl^- SO_4^{2-} TN EC S	8	0.601
pH MC Ca Cl^- SO_4^{2-} TN EC S NH_4^+-N	9	0.592
pH MC Ca Cl^- SO_4^{2-} TN EC S TP NH_4^+-N	10	0.581
pH MC Ca K Cl^- SO_4^{2-} TN EC S TP NH_4^+-N	11	0.575
pH MC Ca K Na Cl^- SO_4^{2-} TN EC S TP NH_4^+-N	12	0.568
pH MC Al Ca K Na Cl^- SO_4^{2-} TN EC S TP NH_4^+-N	13	0.559
pH MC Al Ca Cu K Na Cl^- SO_4^{2-} TN EC S TP NH_4^+-N	14	0.540
pH MC Al Ca Cu K Mg Na Cl^- SO_4^{2-} TN EC S TP NH_4^+-N	15	0.520
pH MC Al Ca Cu K Mg Na Cl^- SO_4^{2-} TN TOC EC S TP NH_4^+-N	16	0.494
pH MC Al Ca Cu K Mg Mn Na Cl^- SO_4^{2-} TN TOC EC S TP NH_4^+-N	17	0.474

Table S8. Canonical correspondence analysis (CCA) of microbial community composition data and main physicochemical parameters

Name	Explains %	Pseudo-F	P
EC	7.2	3.3	0.002
pH	6.7	3.1	0.002
SO_4^{2-}	5.8	2.6	0.002
Fe	5.6	2.6	0.002
S	5.5	2.5	0.002
Ca	5.1	2.3	0.002
TP	3.9	1.7	0.002
TN	3.7	1.7	0.002
TOC	3.7	1.7	0.004
Mg	3.3	1.4	0.012
MC	3.2	1.4	0.014
K	3.1	1.4	0.016
Cl^-	3	1.3	0.022
NH_4^+-N	2.9	1.3	0.012
Cu	2.4	1.1	0.286
Na	2.4	1	0.242
Al	1.9	0.8	0.938
Mn	1.8	0.8	0.834

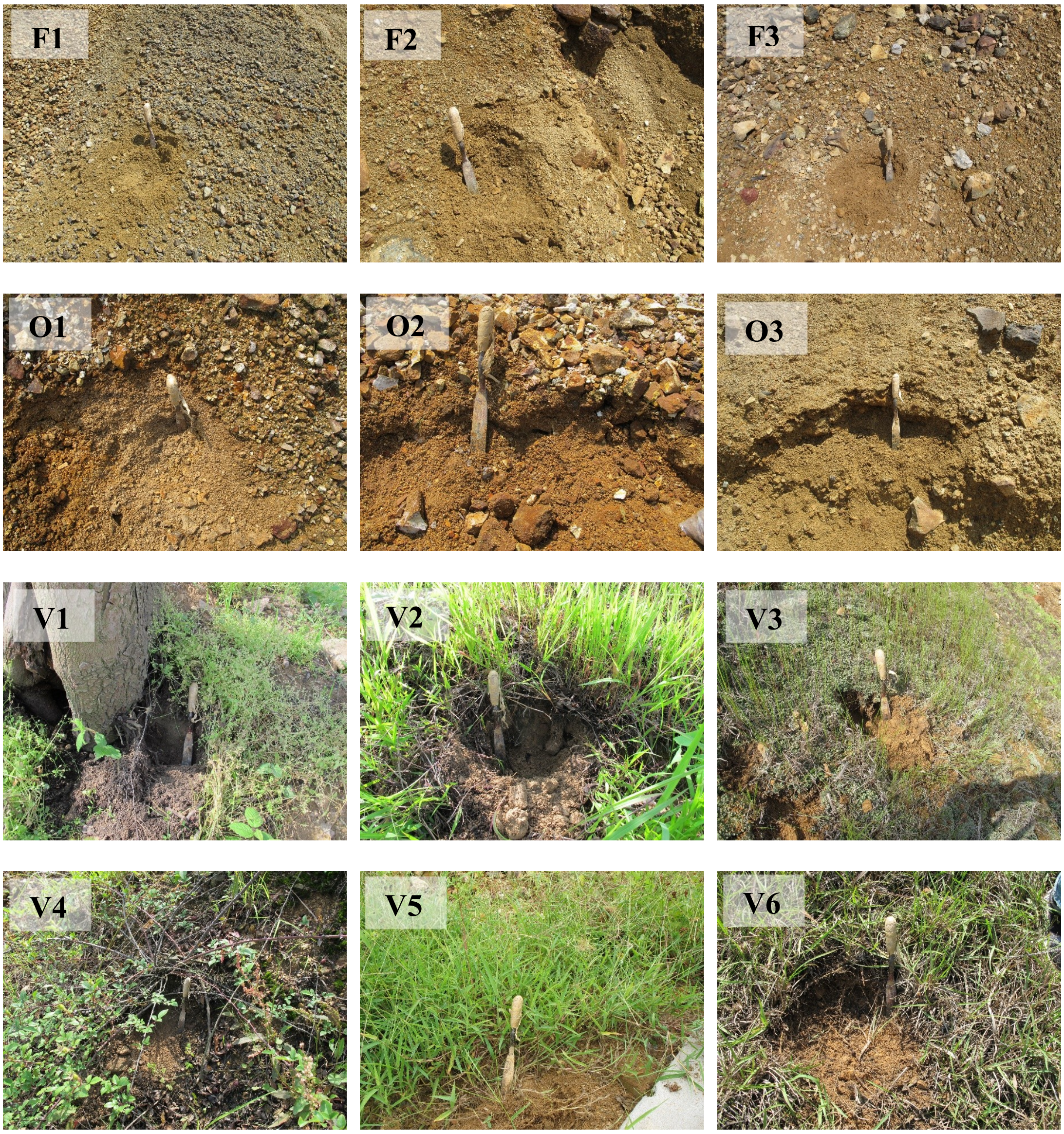

Figure S1. A catalog of selected photos from mine soils sampling sites in the dump of the Nanshan Iron Mine in Anhui Province, China.

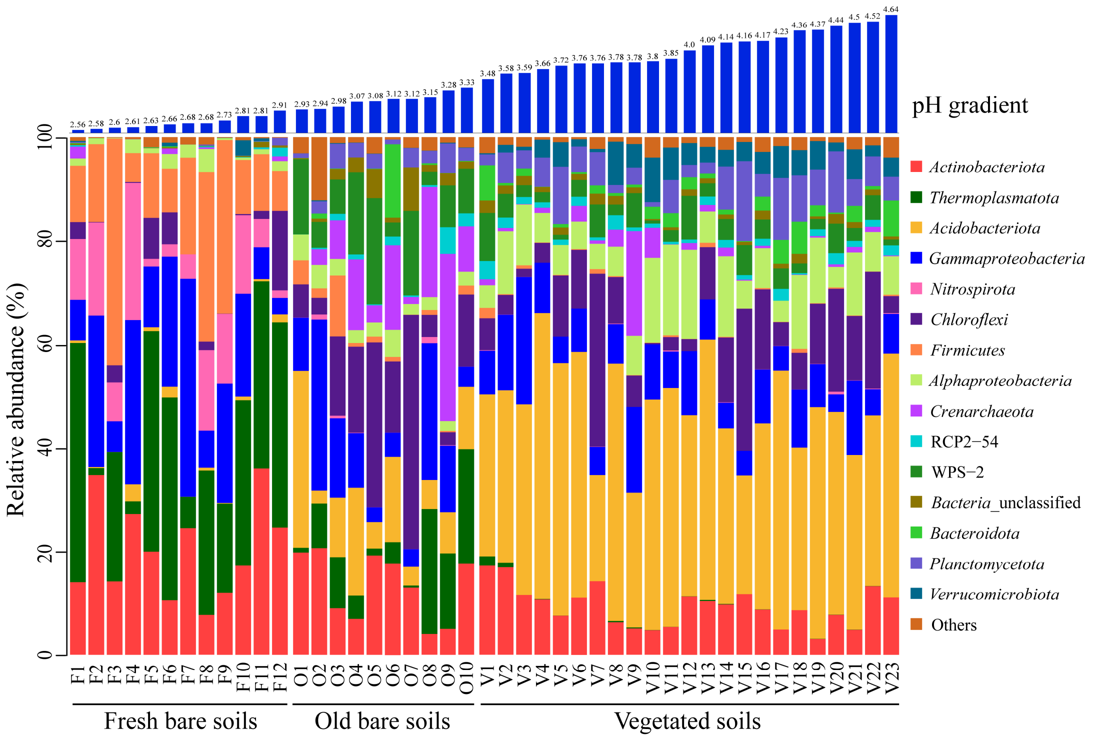

Figure S2. The relative abundance of major phyla/classes along a pH gradient in the 3 types of mine soil samples.

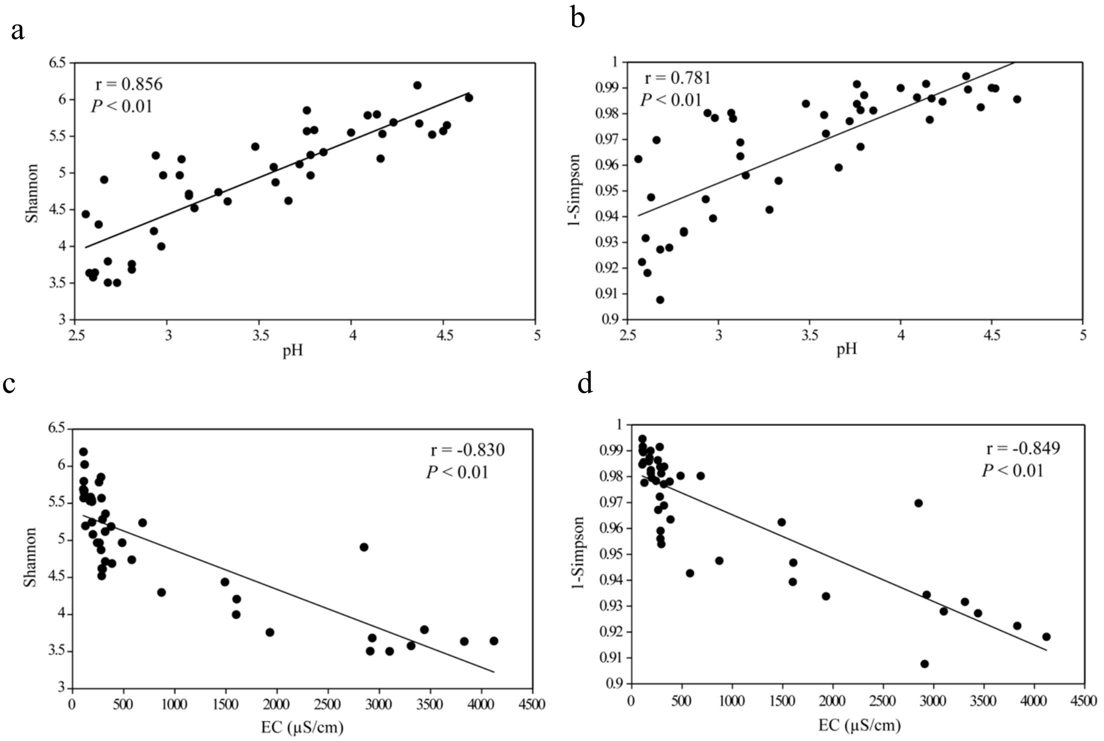

Figure S3. Correlation relationship between physicochemical parameters and diversity indices, pH with (a) Shannon and (b) 1-Simpson, EC with (c) Shannon and (d) 1-Simpson.

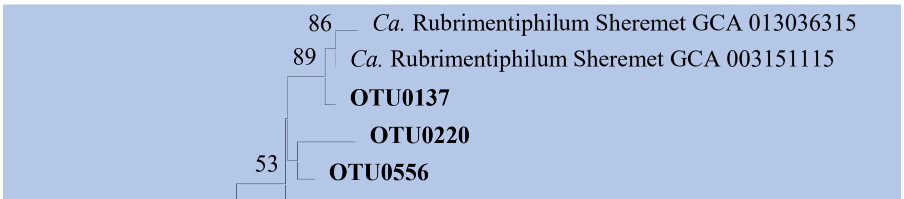

Figure S4. Phylogenetic tree based on 16S rRNA gene sequences of WPS-2 from metagenomic assembled genome and the mine soils. The 16S rRNA gene sequences obtained in this study are in bold characters, 38% of which were clustered with *Ca.* Hemerobacter limicola, a photo/chemo-autotrophic species.
